# Impact of Children’s Presence on Police Responses to Domestic Violence Call Situations

**DOI:** 10.1177/10775595221147315

**Published:** 2022-12-21

**Authors:** Angela Hovey, BJ Rye, Evan George, Susan Scott, Lori Chambers

**Affiliations:** 1 School of Social Work, 7890 Lakehead University, Orillia, ON, Canada; 2 Psychology, St. Jerome's University, University of Waterloo, Waterloo, ON, Canada; 3 Gender and Women's Studies, Lakehead University, Thunder Bay, ON, Canada

**Keywords:** domestic/intimate partner, violence, intimate partner violence, exposure to domestic violence, child

## Abstract

The link between children exposed to intimate partner violence (IPV) and child maltreatment is well established; however, less is known about the impact children’s presence may have on domestic violence disputes. This study investigated the role of children’s presence in IPV police calls using data (*N* = 2709) from supplementary reports provided by an Ontario, Canada police force, one-third of which led to criminal charges (*n* = 909). When children were present: charges were less likely to be laid; the accused’s emotional state was more likely to be rated by police as calm and the accused was less likely to be identified as using alcohol and/or drugs at the time of the call; and victim support interventions were more likely to be offered and accepted. Findings were considered in the context of when charges were laid versus not laid. Implications for police and support service interventions were discussed.

## Introduction

Children who are exposed to intimate partner violence (IPV) are more likely to be at risk for other maltreatment types (Campbell et al., 2020; Chan, 2011). The impact of IPV exposure on children and the link to child maltreatment is well established (Black et al., 2008; Chan, 2011; Edleson, 1999; Fallon et al., 2020; Kimball, 2016; Noble-Carr et al., 2020). Thus, IPV incidents with children present are treated seriously by authorities (Roark et al., 2017; Victor et al., 2019). Child protection laws in Ontario, Canada have a stringent duty-to-report requirement to protect children at risk of maltreatment (e.g., physical, sexual, neglect; Government of Ontario, 2018). This includes reporting children’s exposure to IPV as a potential risk of emotional harm, which may or may not result in further intervention by the child protection agency (Ontario Association of Children’s Aid Societies, 2021). Police are often the first responders to IPV incidents and are the main source of referral to child welfare authorities (Black et al., 2020).

Canadian police respond to a large proportion of non-criminal calls (i.e., 50%–80% of calls resulting in no charges being laid) with domestic disputes being among the top reasons for such calls for service (Conor et al., 2019). In the 1980s, Canada shifted to mandated police intervention for domestic disputes and mandatory charging policies to remove victim responsibility regarding formal complaints (Nikolova et al., 2021). Therefore, police in Canada must investigate every call dispatched and classified as an IPV situation and have no discretion in charging when there is evidence of a crime having been committed (e.g., assault by one partner of the other). In Ontario, Canada, they must also complete a requisite supplementary report specific to IPV (Ministry of the Solicitor General, 2000). In some jurisdictions, these supplementary reports are dependent on the type of investigation (i.e., when charges are laid or not laid). A proportion of these criminal and non-criminal designated calls involve children being present with varying degrees of involvement (i.e., witnessing, hearing, directly involved, or directly assaulted).

IPV situations may have different features because of the presence or absence of children, which could impact interventions by the police. Previous research tended to focus on IPV police calls when charges were laid or arrests occurred (Roark et al., 2017; Swerin et al., 2018) or provided limited analysis in relation to children’s presence (Campbell et al., 2020). The current study analyzes the supplementary report data from a set of IPV calls investigated by Ontario police both when charges were laid (criminal) and when charges were not laid (non-criminal). This study expands upon the limited previous research using in situ police call data pertaining to IPV situations by examining when children were present in both criminal and non-criminal types of incidents; these are conditions that are absent in the current literature. Specifically, this study explores the extent to which children’s presence during police response to IPV calls relate to (1) differences in whether or not charges were laid (including charge frequency and severity); (2) involved persons’ characteristics (i.e., gender, emotional state, and substance use); (3) risk factors identified by police; and (4) police support provisions.

### Police Response to IPV Calls and Children’s Presence

Children’s presence during police response to IPV call incidents ranged from 32% to 59% (Campbell et al., 2020; Fantuzzo & Fusco, 2007; Fusco & Fantuzzo, 2009; Nordham & Pritchard, 2018; Swerin et al., 2018). Different approaches to data collection make comparative conclusions difficult. Most data were based on substantiated events resulting in arrests or charges laid (Fantuzzo & Fusco, 2007; Fusco & Fantuzzo, 2009; Roark et al., 2017; Swerin et al., 2018). Some data was collected via tools designed specifically for the purpose of research (Fantuzzo & Fusco, 2007; Fusco & Fantuzzo, 2009). Other data was part of a pre-existing police database (Campbell et al., 2020; Nordham & Pritchard, 2018; Roark et al., 2017; Swerin et al., 2018).

Arresting or charging decisions in relation to children were examined in some studies. For example, Nordham and Pritchard’s (2018) analysis included incidents in which children were the aggressor towards parents or adults. Roark et al. (2017) examined IPV arrest incidents only when children were present. They found 45% of the incidents resulted in *child abuse charges* laid based on children witnessing, hearing, and/or being directly assaulted. Swerin et al. (2018) found the odds of an arrest decreased when children were present. In addition, the severity and frequency of charges related to IPV incidents indicate a greater likelihood of future violence, including violence towards children (Jaffe et al., 2015). Although the Canadian government collects family violence data, charges pertaining to child victims are presented separately from adult IPV victims. The IPV victim charge data analysis does not include if children were present (Burczycka et al., 2018). Therefore, understanding if children’s presence has a relationship to charging decisions—including frequency and severity of charges laid—in IPV situations could provide further insights into police response.

The police databases used also differed in type of information available or studied beyond arrests or charges. Some used detailed police incident reports (Nordham & Pritchard, 2018; Swerin et al., 2018), while others used only supplementary report data (Campbell et al., 2020). Police response to IPV calls commonly requires the completion of supplementary reports specific to IPV risk factors and other pertinent information that contributed to the IPV call. Campbell et al. (2020) found that children were present or household members in 59% of the reports; however, children’s presence was not analyzed with any other variables. Police supplementary reports are a rich, yet varied, secondary source of information. These reports can provide contextual variables—like risk factors, characteristics of the involved adults, and how police intervene—furthering understanding of IPV incidents when children are present.

### Involved Persons’ Characteristics and Risk Factors

IPV is most commonly men perpetrating violence on women. Men represent over 80% of those charged during police reported IPV incidents (Burczycka et al., 2018; Campbell et al., 2020; Fusco & Fantuzzo, 2009; Roark et al., 2017; Swerin et al., 2018); thus, there is a much greater likelihood of the abuser being a man. However, there is a proportion of women charged or arrested during IPV incidents. When a larger proportion of women are charged, it has been due to charging requirements where both parties were identified as aggressors (e.g., Fusco & Fantuzzo’s study had 27% of women charged, with 14% involving dual charges). Swerin et al. (2018) found that being a woman aggressor significantly increased the odds of an arrest. In studying contexts of women’s arrests, Li et al. (2015) found that some women’s rationale for using violence was in self-defense or to protect their children. Nonetheless, although women are more likely to be victims of IPV, a smaller proportion of women are charged or arrested for IPV. Far less is known about whether or not children being present impacts these situations.

Risk factors for child maltreatment and for adult victims of IPV have some commonality but do differ. However, research has indicated that when adult victims are at risk of IPV or domestic homicide, children are also at risk (Jaffe et al., 2015). Police-based and child welfare studies identified some common risk factors related to IPV and child maltreatment; these involved-adult factors include: substance abuse (Campbell et al., 2020; Fusco & Fantuzzo, 2009; Jaffe et al., 2015; Lawson, 2019; Mason, 2003; Nikolova et al., 2021; Roark et al., 2017; Sijtsema et al., 2020; Victor et al., 2019; Yoo et al., 2015), separation and divorce (Campbell et al., 2020; Jaffe et al., 2015; Sijtsema et al., 2020), mental health issues (Jaffe et al., 2015; Lawson, 2019; Nikolova et al., 2021; Sijtsema et al., 2020; Yoo et al., 2015), and social isolation (Lawson, 2019; Nikolova et al., 2021; Sijtsema et al., 2020; Victor et al., 2019; Yoo et al., 2015).

Findings related to substance use and mental health issues were mixed. Fusco and Fantuzzo (2009) found that substance use by either involved person was less likely with children present. Caregiver/parent substance use, when associated with IPV, predicted substantiation of child welfare cases and significantly increased the odds of child protection service interventions (e.g., in-home support or out-of-home placements; Lawson, 2019; Victor et al., 2019). IPV abusers’ substance use increased risk of violence for children (Jaffe et al., 2015; Mason, 2003; Roark et al., 2017) as did IPV victims or primary caregivers’ substance use (Yoo et al., 2015). Not necessarily predictive of intervention or charges, mental health issues are commonly identified as a risk factor with victims and perpetrators of IPV and/or child maltreatment. The impact of children’s presence during IPV incidents on associated risk factors would provide further understanding of their IPV exposure. Further, the emotional state of the involved persons at the time of the police response to the call has not been examined in police-based studies and could provide important contextual information about IPV call incidents.

### Police Offered Support

Police response to IPV calls involves connecting victims and children to support services. Some support involves taking IPV victims directly to a place of safety, such as women’s shelters, providing a crisis line number, or offering them referrals to victim services. Victims may or may not accept this type of support. Meyer (2010) found that about 75% of victims will seek help but most do so informally from family or friends. A much smaller proportion will seek formal help from police (19%) or seek refuge in a shelter (under 3%); however, the key predictor for victim help-seeking from police or refuge was when children witnessed the IPV (Meyer, 2010). While provision of victim supports was low (i.e., 10% of cases), Swerin et al. (2018) also found that children’s presence during IPV-arrest incidents increased the odds of police providing supports/referrals to the victim. In contrast, Goodson et al. (2022) found just over 70% of victims were provided with service referrals by police, although children’s presence had no impact on service referral provision. Hatten and Moore (2010) recommended a pamphlet with victim services information be created to ensure supports are offered to every victim after they found that police were less likely to contact victim services depending on situational characteristics (e.g., victim substance use, male victim).

Police tend to rely on child protection services to manage the support and safety of children (Saxton et al., 2020). As noted earlier, Ontario police are required to notify child protection services when responding to IPV calls where children are present regardless of whether or not any charges are laid (Government of Ontario, 2018; Nikolova, 2021). Thus, child protection agencies are inundated with these types of referrals, but do not have the capacity to provide the needed services for lower-risk IPV-exposure referrals (Black et al., 2020). Police notifications tend to lack sufficient child-related details during domestic disputes (Stanley et al., 2011). Police usually focus on speaking with the adults unless the child had been directly assaulted or physically hurt (Richardson-Foster et al., 2012). More effective collaboration with police about the risk and context of the IPV referrals is an important opportunity to improve safety planning and intervention (Olszowy et al., 2020).

In summary, children’s experiences with IPV may not be captured as well as they could be by police (Elliffe & Holt, 2019). The police supplementary form includes additional IPV incident information (e.g., risk factors, involved adult characteristics, how police intervene with children and victims) that contribute further insights about the impact of children’s presence during IPV incidents. Most IPV police response research has examined children’s presence when charges or arrests occurred. Given the substantial proportion of non-criminal IPV calls, this has produced a very limited understanding of children’s presence during non-criminal IPV situations.

### Current Study

Using police supplementary report data, this study examined the role of the presence or absence of children during police calls in relation to several aspects of the IPV situations. Specifically, children noted as present during IPV calls was explored in relation to characteristics of the adults involved (i.e., gender, emotional states, and substance use), and police-assessed risk factor questions, as well as victim support service offers and safety interventions noted by police. Whether criminal charges were laid or not laid was evaluated as a potential moderating factor of children’s IPV call presence. Specifically, was there a relationship between children’s presence at IPV calls and: (1) Charges being laid (and differences in number and severity of charges laid); (2) Involved person characteristics (i.e., gender, emotional state, and substance use) stratified by charges laid or not; (3) Risk factors; and (4) Police support offers and victim’s acceptance of support (stratified by charges laid or not)?

## Method

In some Ontario jurisdictions, police must complete one of two requisite IPV ancillary report forms dependent on the type of investigation: when criminal charges are laid (CC); or when no criminal charges are laid (NonCC). These forms were not designed for research purposes nor are they the complete record of an official police report; they are supplementary and serve as a checklist for the investigation and monitoring of follow-up. Accordingly, we conducted a secondary analysis of real-world data collected, without the intent of conducting research, by a police service in a small-sized city with rural areas, in Ontario, Canada. Specific research questions addressed were constrained by the information contained in the supplementary forms and the associated data.

### Measurement

The unit of analysis was police calls—rather than couples or individuals or cases—that occurred within the time frame of January 2011 to December 2013 that were resolved prior to extraction of the data. Information collected for each call included: responses regarding the presence of children; if charges were laid by police, the precise charges; genders and roles of involved persons (“accused” and “victim” are used but the accused equivalent is “respondent” and the victim equivalent is “complainant” for NonCC calls); an emotional state checklist; alcohol and/or drug use by involved persons at the time of the call; a checklist of risk factor questions; and victim support offerings and acceptance. The forms did not include any data pertaining to age or race. Police had an option to list ages of children present; however, this was completed inconsistently, and thus, was not reliable for analysis. Almost all information collected was nominal or dichotomous in nature (i.e., yes/no/unknown or indicated/not indicated in the tick-boxes or checklists). Consequently, most analyses involve non-parametric statistics.

### Sample Description

Of 2709 IPV calls analyzed, 66.5% (*N* = 1800) did *not* result in criminal charges being laid, while about a third (i.e., 33.6%; *N* = 909) resulted in charges. Slightly over a third (33.9%; *n* = 917) of all IPV calls reported that children were present. In terms of gender of involved persons, 68.4% (*n* = 1852) of those accused were male; while 69.0% (*n* = 1869) of the victims were female. When children were present and charges were laid, number of charges noted ranged from 0 to 23.

### Analysis and Variables

Whether children were present or not at an IPV call was the critical predictor in each statistical analysis. The significance of the relationship between presence of children and other key variables (e.g., substance use, risk factor presence) was assessed using chi-square tests, while strength of the relationship was described using the phi (φ) correlation statistic. Odds ratios and risk ratios were also presented. Another critical variable of interest was whether or not charges were laid, which was assessed as a potential moderator of the relationship between presence of children and other variables of interest (e.g., emotional state of the accused) for many of the assessments. Therefore, charges laid or not was a stratification variable.

## Results

### Children’s Presence and Charges

The initial premise of most of the analyses was that children’s presence (or not) was moderated by whether charges were laid or not. For stratification, a relationship between the presence of children and criminal charge status had to be established. Whether or not children were present at an IPV call had a systematic relationship with whether or not charges were laid (φ = −.05). When children were present compared with when children were NOT present, fewer charges were laid (30.5% vs. 35.3%; *z* = 2.64, *p* < .01). There was a 14.3% decrease in the likelihood of a charge laid when children were present (see Table 1: Charges Laid). When children were present or not, there were no differences in the number of charges laid (*M* = 2.02 [SD = 1.98, *n* = 277] vs. *M* = 1.81 [SD = 1.39, *n* = 632], *t*(399) = −1.65, *ns*, η_p_^2^ = .00). The seriousness of the charges laid, based on the charge type (i.e., summary or indictable) and maximum penalty possible for the charge, did not differ as a function of the presence of children (χ^2^(6) = 1.90, *ns*).

**Table 1.** Relationship of Children’s Presence and Outcome Variables (with no Stratification).

Outcome Variable		Children		χ^2^(1)	φ	OR [95% CI]
		Present % (*n*)	NOT Present % (*n*)			
Charges laid	YES	10.2 (277)	23.3 (632)	6.97^**^	−.05	.79 [.67, .94]
	NO	23.6 (640)	42.8 (1160)			
Accused calm state	YES	15.2 (413)	26.7 (723)	5.49^*^	.05	1.21 [1.02, 1.42]
	NO	18.6 (504)	39.5 (1069)			
Accused substance use	YES	5.5 (149)	12.9 (349)	4.21^*^	−.04	.80 [.65, .99]
	NO	28.3 (768)	53.3 (1443)			

*Note. N* = 2709.

^*^p < .05. ^**^p < .01.

### Children’s Presence and Involved Person Characteristics

#### Accused Gender

More males than females (68.5% vs. 31.5%, *z* = 19.25, *p* < .001) were the accused for the IPV calls. Gender of accused differed depending on whether or not charges were laid (χ^2^(1) = 10.06, *p* = .002, φ = .06; OR: .75, 95% CI [.63, .90]). Specifically, when charges were laid, there was a 21.1% greater likelihood of the accused being male. When no charges were laid, there was an 8.8% decreased likelihood of the accused being male.

The relationship between children’s presence and accused gender when charges were laid was significant (φ = .09). When children were present at the call, there was a 29.5% decrease in the likelihood that the accused was female and a 12.8% increased likelihood that the accused was male. However, there was no relationship between children’s presence and accused gender (φ = .03) when there were no charges laid (see Table 2). Factoring in the moderating effect of whether or not charges were laid, when children were present, the likelihood of an accused being female decreased by 13.8% and increased for an accused man by 6.8% (φ = .04). These latter statistics are more accurate, yet lower, because they incorporate the moderation effects of whether or not charges were laid.

**Table 2.** Relationship of Children’s Presence and Gender Variables Stratified by Charges Laid.

Outcome and Stratification Variables		Children		χ^2^(1)^a^	φ	OR [95% CI]
		Present % (*n*)	NOT Present % (*n*)			
Accused Gender						
CC	Woman	6.5 (59)	21.0 (191)	5.64^*^	.04	1.24 [1.04, 1.48]
	Man	24.0 (218)	48.5 (441)			
NonCC	Woman	11.4 (204)	22.1 (397)		
	Man	24.3 (436)	42.2 (757)		
Victim gender						
CC	Woman	24.3 (221)	48.6 (442)	9.46^**^	−.10	.59 [.42, .83]
	Man	6.2 (56)	20.9 (190)			
NonCC	Woman	24.4 (439)	42.6 (767)	1.24^*ns*^	−.03	.89 [.72, 1.09]
	Man	11.1 (200)	21.8 (393)			

*Note.* Accused gender missing for 6 non-criminal code calls resulting in a slightly different *N* (2703 vs. 2709).

^a^Mantel-Haenszel χ^2^ for Accused Gender. Test of Homogeneity of OR for Victim Gender was significant; therefore, Mantel-Haenszel was not appropriate and each stratum was considered separately.

^*^p < .05. ^**^p < .01.

#### Victim Gender

The victims were male in 31.0% of calls, while females were the victim in 69.0% of calls (*z =* 19.78, *p* < .001). The underlying test assumption comparing the relationship of children’s presence and victim gender stratified by charges laid was violated so it was not interpreted. However, examining the relationship for each isolated stratum suggested there was a relationship between children’s presence and victim gender when charges were laid (φ = −.10). That is, the likelihood of a female victim increased by 14.1%, while the likelihood of a male victim decreased by 32.8% when children were present and charges laid. When no charges were laid, children’s presence and victim gender had no relationship (see Table 2).

#### Emotional State

“Calm” was chosen to represent emotional state because it correlated with all other emotional state adjectives (i.e., angry, upset, crying, nervous, afraid, hysterical, apologetic, threatening). The emotional states of involved people could be separated by victim and accused; however, there was no relationship between children’s presence and the victim’s emotional state regardless of whether charges were laid or not (all χ^2^(1) < 1.00, *ns*).

The emotional state of the accused was not significantly different as a function of children present when charges were laid (χ^2^(1) = 3.00, *ns*, φ = .06) nor when charges were not laid (χ^2^(1) = 1.50, *ns*, φ = .03) and there was no moderating effect of charges laid or not (Mantel-Haenszel: χ^2^(1) = 3.69, *ns*). However, when the data were collapsed across charges laid and not laid, the relationship between accused emotional state and children present was weak but significant (φ = .05; see Table 1: Accused Calm State). While the accused was judged to be calm a substantial amount of the time (41.9%), most of the time (58.1%; *z* = 8.40, *p* < .001) the accused was *not* marked as calm. There was an 11.6% increased likelihood that the accused would be rated as calm when children were present.

#### Substance Use

The substance use variable included the categories of alcohol use and/or drug use as an identified state of the involved persons noted by the police at the time of the call. While overall substance use was relatively low, the accused was more likely to be indicated as using substances compared to the victim (18.4% vs 10.3%; *z* = 8.49, *p* < .001). Whether or not charges were laid did not moderate the relationship between the presence of children and the substance use of the accused (Mantel-Haenszel: χ^2^(1) = 2.36, *ns*). Collapsing across charges laid or not laid, there was a small but significant relationship between children’s presence and accused substance use (φ = −.04). There was a 16.6% lesser likelihood that an accused was using substances when children were present (see Table 1: Accused Substance Use).

There were significant relationships between children’s presence and victim’s substance use—both for when charges were laid and not laid. However, the odds ratios (OR) were *not* the same as a function of whether or not charges were laid (Test of Homogeneity of OR: Tarone’s: χ^2^(1) = 6.37, *p* = .012). Because of this underlying test assumption violation, the Mantel-Haenszel statistic was not appropriate and each stratum—charges laid or not laid—was considered separately (see Table 3). When charges were laid, there was a significant and strong relationship between children’s presence and victim’s substance use (φ = −.16). There was a 73.6% decrease in the likelihood that the victim was using substances when children were present at the call. When charges were *not* laid, there was also a significant, yet weaker, relationship between children’s presence and victim’s substance use (φ = −.07). Similar to when charges were laid, there was a 38.2% decrease in the likelihood that the victim was using substances when children were present.

**Table 3.** Relationship Between Children’s Presence and Victim Outcomes Stratified by Charges Laid.

Outcome and Stratification Variables		Children Present		χ^2^(1)^a^	φ	OR [95% CI]
		Present % (*n*)	NOT Present % (*n*)			
Victim Substance Use						
CC	YES	1.2 (11)	10.5 (95)	22.87^***^	−.16	.23 [.12, .44]
	NO	29.3 (226)	59.3 (537)		
NonCC	YES	2.4 (44)	7.2 (129)	8.56^**^	−.07	.59 [.41, .84]
	NO	33.1 (596)	57.3 (1031)		
Offer to contact victim services						
CC	YES	29.6 (269)	62.9 (569)	13.41^***^	.12	3.72 [1.76, 7.88]
	NO	0.9 (8)	6.9 (63)		
NonCC	YES	29.0 (522)	47.9 (862)	12.21^***^	.08	1.53 [1.20, 1.94]
	NO	6.6 (118)	16.6 (298)		
Victim services offer accepted by victim						
CC	YES	4.8 (40)	7.4 (62)	18.19^***^	.09	1.78 [1.37, 2.32]
	NO	27.3 (229)	60.5 (507)		
NonCC	YES	5.9 (82)	5.2 (72)		
	NO	31.8 (440)	57.1 (790)		
Victim taken to a place of Safety/Women’s shelter						
CC	YES	14.6 (133)	25.9 (235)	22.17^***^	.09	1.44 [1.27, 1.76]
	NO	15.8 (144)	43.7 (397)		
NonCC	YES	13.4 (242)	18.9 (341)		
	NO	22.1 (398)	45.5 (819)		

*Note. N*_CC_ = 909, *N*_*NonCC*_ = 1800 except for Victim Services Offer Accepted where *N*_*CC*_ = 838, *N*_NonCC_ = 1384.

a Mantel-Haenszel χ^2^ for Victim Substance Use and Offer to Contact Victim Services. Tests of Homogeneity of OR for Victim Services Offer Accepted and Victim Taken to a Place of Safety were significant; therefore, Mantel-Haenszel tests were not appropriate and each stratum was considered separately.

^*^p < .05. ^**^p < .01. ^***^p < .001.

### Children’s Presence and Risk Factors

Each supplementary form included a unique list of risk factor questions (i.e., CC: 20 questions; NonCC: 16 questions). Because of the differences and wording of these questions, the risk factors could not be compared directly (i.e., no stratification analysis was possible). However, a few observations were warranted between the two sets of risk factors. There was significantly greater risk factor endorsement for investigations resulting in criminal charges; 65% (13 of 20) of risk factors were endorsed at over 20% whereas only 31% (5 of 16) were endorsed at over 20% when no charges were laid (*z* = 2.01, *p* < .05). Next, the significant relationships between children’s presence and risk factor endorsement were relatively stronger when charges were laid compared to when no charges laid (i.e., φs _CC_ = .07-.22 vs. φs _NonCC_ = .05-.08; however, the proportion of significant relationships did not differ, 30.0% vs. 31.3%, *z* = −0.08, *ns*).

#### Charges Laid Risk Factors

The most notable difference when children were present relative to when children were not present was a 205.7% increased likelihood that the police noted there was *a recent change in contact between the children and the accused*. The risk factor of *the accused experienced any unusually high stress recently* was significantly related to children’s presence such that, when children were present, there was a 38.1% increased likelihood that this risk factor was endorsed. There was an approximate 45% increase in the likelihood that the risk factors of *the accused threatened or destroyed the victim’s property* and *a recent escalation in frequency or severity of assaults/threats against the victim* were endorsed when children were present (44.9% and 46.3%, respectively). When children were present, the likelihood of the risk factor *the victim fears that the accused will continue assaults, seriously injure, or kill her/him/children* being endorsed increased by a third. Finally, when children were present, *the accused’s personality features anger, impulsiveness, or poor behavior control* risk factor endorsement increased in likelihood by 16.4%. In short, children’s presence significantly predicted increased endorsement likelihood of these particularly volatile risk-factor circumstances (see Table 4).

**Table 4.** Relationships between Children’s Presence and Significant Risk Factors.

Risk Factors		Children		χ^2^(1)	φ	OR [95% CI]
		Present % (*n*)	NOT Present % (*n*)			
CC - Accused (*N* = 909)						
Contact change with children	YES	7.4 (67)	5.5 (50)	45.50^***^	.22	3.71 [2.49, 5.53]
	NO	23.1 (210)	64.0 (582)			
High stress	YES	14.9 (135)	24.5 (223)	14.60^***^	.14	1.74 [1.31, 2.32]
	NO	15.6 (142)	45.0 (409)			
Damaged property	YES	9.6 (87)	15.1 (137)	9.82^**^	.10	1.65 [1.21, 2.27]
	NO	20.9 (190)	54.5 (495)			
Escalation in Conflict	YES	8.3 (75)	15.1 (117)	8.48^**^	.10	1.63 [1.17, 2.28]
	NO	22.2 (202)	56.7 (515)			
Victim fears assault	YES	10.7 (97)	18.3 (166)	7.18^**^	.09	1.51 [1.12, 2.05]
	NO	19.8 (180)	51.3 (466)			
Externalizing behaviors	YES	16.7 (152)	32.8 (298)	4.59^*^	.07	1.36 [1.03, 1.81]
	NO	13.8 (125)	36.7 (334)			
NonCC – Either involved person (*N* = 1800)						
More Conflict in Relationship	YES	12.2 (220)	17.1 (307)	12.46***	.08	1.46 [1.18, 1.79]
	NO	23.3 (420)	47.4 (853)			
Stalking	YES	1.9 (34)	5.7 (102)	7.15^**^	−.06	0.58 [.39, .87]
	NO	33.7 (606)	58.8 (1058)			
Violence to one another	YES	5.8 (105)	7.8 (141)	6.32^*^	.06	1.42 [1.08, 1.86]
	NO	29.7 (535)	56.6 (1019)			
Externalizing behaviors	YES	9.1 (164)	13.8 (248)	4.21^*^	.05	1.27 [1.01, 1.59]
	NO	26.5 (476)	50.7 (912)			
Fears assault	YES	2.4 (48)	3.3 (60)	3.96^*^	.05	1.42 [1.08, 1.59]
	NO	32.9 (592)	61.1 (1100)			

*Note.* CC and NonCC supplemental forms did not contain the same risk factors and referred to different targets (i.e., accused versus either/both involved parties) making a stratified analysis impossible.

^*^p < .05. ^**^p < .01. ^***^p < .001.

#### No Charges Laid Risk Factors

There were five significant relationships between children’s presence and risk factor endorsement when no charges were laid. When children were present, there was an increased likelihood that the following risk factors were endorsed: *relationship has become more conflictual* (increased likelihood of endorsement by 29.9%); *either involved person demonstrated violence toward others* (35.0%); *either person’s personality feature anger, impulsiveness, or poor behavior control* (19.9%); and *either person fear the other will assault, injure or kill her/him/children* (45.0%). In contrast, *either involved person demonstrated stalking behavior* decreased in endorsement likelihood by 39.6% when children were present, but this must be taken in the context of the overall low endorsement of the stalking risk factor (endorsed for 7.6% of calls). See Table 4 for the contingency table and χ^2^ statistics associated with the significant risk factors and children’s presence relationships.

### Children’s Presence and Police Intervention with Victims

Victim-based supports were offered or provided by police during their call response. Two police action interventions and victim responses were analyzed. First, the offer to contact victim services and victim’s acceptance of the offer involved the police offering to make a referral to the local victim services agency for follow-up support and whether or not this offer was accepted. Second, the victim being taken to a place of safety involved police transporting or arranging for victims to be taken to a safe location such as a relative or friend’s home or a women’s shelter.

#### Offer to Contact Victim Services and Victim Acceptance of the Offer

Police offered to contact victim services significantly more often when charges were laid than when charges were not laid (92.2% vs. 76.9%; *z* = 9.79, *p* < .001). Police were more likely to offer to contact victim services when children were present compared to when children were not present (86.3% vs. 79.9%; *z* = 4.11, *p* < .001). There were significant relationships between children being present and police offers to contact victim services when charges were laid (φ = .12) and when charges were not laid (φ = .08). The underlying assumption of homogeneity of odds ratios was violated (Tarone’s: χ^2^(1) = 5.17, *p* = .023). Thus, each stratum— charges laid or not laid—was considered separately (see Table 3).

When charges were laid and children were present, there was a 7.9% higher likelihood that police would offer to call victim services; with no children, there was a 24.5% decreased likelihood of an offer to call victim services. When no charges were laid and children were present, there was a 9.8% higher likelihood that police would offer to call victim services. When charges were not laid and there were no children present, there was a 14.2% decreased likelihood of offering to call victim services. In short, there was an overall high rate of police offering to call victim services when children were present in any IPV calls. However, a much lower proportion of victims accepted the offer.

Overall, acceptance of offers to contact victim services was low (11.5%). Acceptance was significantly different depending on whether or not children were present and whether or not charges were laid (φ = .09). The weighted average across both CC and NonCC calls suggest that, if children were present, there was a 60.9% greater likelihood that the victim would accept an offer of a call to victim services. Considering whether or not charges were laid separately, when children were present, the increased likelihood of acceptance was 36.5% for CC calls and 88.1% for NonCC calls (see Table 3).

#### Victim Taken to a Place of Safety

Collectively, victims were taken to place of safety in 35.1% of calls (i.e., more so for CC = 40.5% vs. NonCC = 32.4%; *z* = 4.17, *p* < .001). When children were present, regardless of whether or not charges were laid, 40.9% of calls resulted in victims being taken to a place of safety in contrast to 32.1% when no children were present (*z* = 4.52, *p* < .001). The relationship between children’s presence and victim being taking to a place of safety was significant considering the context of the stratifying effect of whether or not charges were laid (φ = .09). When children were present when charges were laid, the likelihood of a victim being taken to a place of safety increased by 28.8% and decreased in likelihood by 13.5% when no charges were laid (see Table 3).

## Discussion

The aim of this study was to investigate if characteristics of IPV calls were different as a function of children’s presence. The analysis of police supplementary forms suggested that there were some significant differences in police response when children were present at calls. The findings were mixed regarding charges being laid. That is, charges were less likely to be laid when children were present, consistent with Swerin et al.’s (2018) findings of decreased odds of an arrest with children’s presence. Arrests in front of children can further traumatize children, add additional stress and emotional trauma (McCormick et al., 2019), and increase children’s anxiety symptoms (Jouriles et al., 2020). Police were recommended to avoid arrests in front of children and to take time to explain in developmentally appropriate language to try to reduce the negative impact (McCormick et al., 2019). However, this study found, when charges were laid, there was no relationship between children’s presence and the seriousness of charges laid or number of charges laid. Other police call studies did not analyze these charge-based variables with children’s presence. Perhaps the IPV situations when children were present involved no evidence of criminal behavior required for charges.

The analysis of the characteristics of the adults involved might offer another possible explanation as to the reduced likelihood that charges were laid when children were present. This sample had a somewhat smaller proportion of men being charged (68.5%) compared to other studies (over 80%; Burczycka et al., 2018; Campbell et al., 2020; Fusco & Fantuzzo, 2009; Roark et al., 2017; Swerin et al., 2018). There was only one real moderating effect of charges laid or not laid pertaining to gender. When children were present and charges were laid, there was a much lower likelihood that the accused was a woman, but there were no accused gender differences when children were present and no charges were laid. These results suggest that the calls represent more stereotypical IPV incidents of male-to-female violence, but IPV situations resulting with no charges represent a more complex situation in relation to gender. Further research is needed to better understand the roles of involved persons’ gender and children’s presence in IPV situations where no charges are laid.

We found other significant characteristics of involved adults when children were present. The accused was more likely to be rated as calm and was less likely to be using substances. Fusco and Fantuzzo (2009) found children were less likely to be exposed to IPV when either the victim or accused was using substances. Similarly, when children were present, the victim was far less likely to be using substances compared to when children were not present; this relationship was strongest, though, when charges were laid. The victims’ lower rates of identified substance use at time of the police call appear similar to other studies (Campbell et al., 2020; Fusco & Fantuzzo, 2009; Roark et al., 2017). However, it is noteworthy that overall substance use was low in this study, differing substantially from findings in other studies of IPV police calls (Campbell et al., 2020; Friend et al., 2011; Fusco & Fantuzzo, 2009; Mason, 2003; Roark et al., 2017). That the accused was more likely to be rated as calm was a surprising finding. Although no other studies examined the emotional states of involved persons, IPV is commonly equated with more emotionality. In general, the results suggest that when children are present at an IPV call, they may act as a *sort-of* protective factor as the adults are demonstrating lower volatility (i.e., less substance use, a calmer demeanor).

A different component of the IPV situation included risk factors assessed by police at the call, which appear to contradict the demonstrated lower volatility. Although the two forms do not allow for direct comparison of risk factors, some common themes emerged between the two: both noted volatile personality behaviors and traits and both involved fear by one partner that the other partner would assault or injure them or the children. A moderately similar set of risk factors involved escalation in assaults or threats against the victim (CC form) and the relationship becoming more conflictual plus the involved persons demonstrated violence toward another (latter two risk factors from the NonCC form). In short, what appears to be common, when children are present versus not present, are elevated risk factors of the accused or respondent demonstrated anger, impulsiveness, or poor behavior control and has threatened or engaged in violent behavior toward the victim/children. Additionally, the victim or complainant has fear of being assault assaulted by their partner. These risk factors appear to contradict the less volatility explanation regarding the impact of children’s presence and might suggest that involved persons may be modifying their behaviors at the time of police call response due to beliefs that they may be treated more harshly when children are present.

Another police response domain investigated was police actions in relation to the victim including offers by the police to contact victim services and taking the victim to a place of safety or women’s shelter. Overall, offers to contact victim services were at a high rate, but when children were present at the call, police were even more likely to make an offer to contact victim services. However, there was a substantially low acceptance rate by victims of this offer. Nonetheless, when children were present, there was a significantly greater likelihood the offer would be accepted, particularly when no charges were laid. Further, the presence of children increased the likelihood that the police would take the victim (and children) to a place of safety/shelter, more so when charges were laid. Although Swerin et al.’s (2018) analysis collapsed types of support interventions into one variable, this study’s results are consistent with Swerin et al.‘s in that children’s presence increased the odds of support interventions by police. However, their data contained a substantially lower proportion of interventions (only 10% versus the current study’s 82% for referral offers and 41% for place of safety).

### Limitations

The big advantage of this study was that it is based on real life data, but that was also its greatest limitation. It is a secondary analysis of data that was reliant on the police who filled out the supplementary form in real life/in situ, and this was not necessarily at the time of the call (e.g., back at the office, at end of shift). The form was clearly not designed for research purposes with yes/no/unknown or checked/not checked options and several narrative boxes that required interpretation. If questions were left blank, the data was presumed unknown or simply not checked; thus, the meaning of missing data could differ. Also, some charges may be indirectly related to the IPV incident response. Although several analyses produced significant relationships, they could be considered negligible. Regardless, the weakness of these relationships must be taken in the context of the dichotomous data; this was not laboratory-controlled data with forms designed specifically for research. The primary unit of analysis, police calls, also limits the data, requiring that each call must be treated as a unique situation rather than using a unit of analysis of a person-based case or couple. Approximately 25% of all calls involved two people who had not been involved in any other calls. In the majority of the calls, including those in which children were present, one or both persons were involved in two or more calls, and often with other partners.

### Implications for Practice

Despite the inconsistencies and poorly designed forms, the results do tell a relatively consistent story, which offers an important contribution to a minimally-researched topic of supplementary police call report content. That *any* systematic relationships or differences from this real-world data were found is suggestive that effects are probably present. The police supplementary forms can provide important information; however, streamlining this data and improving processes for report completion may help with utility of the data. The form information should be easily accessed by responding officers and guide interventions, particularly with repeated calls to the same household. This analysis can be used to help streamline data collected at IPV police calls (e.g., no need to ask about many different emotional states when calm/not calm will suffice). While flawed, these findings are an impetus to conduct more controlled studies. As well, expanding the examination and scope of police call data to other jurisdictions and contexts is needed.

Understanding children’s presence at IPV calls could impact intervention decisions by police and service providers in the call-response moment. For example, child protection workers may secure more substantive information to assess the child protection needs by exploring questions related to volatility before police were called in contrast to how the accused presented (i.e., calm) when police responded. As well, the under-utilization of risk data (and inconsistent completion) has opportunity for improvement. Although the risk data is designed for prediction of future incidents or higher risk cases (Medina Ariza et al., 2016; Seewald et al., 2017), it could be used more effectively in designing appropriate support interventions, specifically in relation to children. Collaborating more effectively by determining key information of relevance for police to share with child protection workers and vice versa may improve the notification system between these mandatory services (Olszowy et al., 2020; Stanley et al., 2011).

Given that approximately 66% of calls result with no charges laid and there appears to be less volatility (e.g., offenders are more likely to be calm and less likely to use substances) during a substantial number of calls in which children are present, integrating social workers or other service providers in these less volatile moments of crisis could bolster the supportive services offered to victims and their children and potentially increase the acceptance of support by victims. Hatten and Moore (2010) found that when victims are offered support, they commonly decline; however, if the support is more readily available in the moment of crisis, the victim may be more willing to engage with support services in ways that could benefit children. Some police services have hired social workers or partnered with agencies to conduct follow-up calls and provide case management in IPV cases for ongoing supportive intervention (Messing & Campbell, 2016; Ward-Lasher et al., 2017).

Although children are not and should not be the primary focus in a police investigation of IPV, the presence of children changes the dynamic of the police call response. Police could benefit from examining how the presence of children can, explicitly or implicitly, impact their actions during call responses. Relevant information concerning children can be extracted from supplementary reports to strengthen communication exchanges and collaboration between police and child protection services. Using the police call data to design more effective and timely support service interventions, particularly for victims and children exposed to IPV, has the potential to reduce the volume of non-criminal IPV calls and risk of child maltreatment.
